# Whole-Genome Sequence of Bacillus subtilis WS1A, a Promising Fish Probiotic Strain Isolated from Marine Sponge of the Bay of Bengal

**DOI:** 10.1128/MRA.00641-20

**Published:** 2020-09-24

**Authors:** M. Mahbubur Rahman, Sulav Indra Paul, Tasmina Akter, Alfred Chin Yen Tay, M. Javed Foysal, M. Tofazzal Islam

**Affiliations:** a Institute of Biotechnology and Genetic Engineering, Bangabandhu Sheikh Mujibur Rahman Agricultural University, Gazipur, Bangladesh; b Department of Fisheries Management, Bangabandhu Sheikh Mujibur Rahman Agricultural University, Gazipur, Bangladesh; c Marshall Centre for Infectious Diseases Research and Training, School of Biomedical Sciences, University of Western Australia, Perth, Western Australia, Australia; d Department of Genetic Engineering and Biotechnology, Shahjalal University of Science and Technology, Sylhet, Bangladesh; e School of Molecular and Life Sciences, Curtin University, Bentley, Western Australia, Australia; University of Maryland School of Medicine

## Abstract

This study reports the draft genome sequence of a promising fish probiotic, Bacillus subtilis strain WS1A, that possesses antimicrobial activity against Aeromonas veronii and suppressed motile *Aeromonas* septicemia in Labeo rohita. The *de novo* assembly resulted in an estimated chromosome size of 4,148,460 bp, with 4,288 open reading frames.

## ANNOUNCEMENT

The Bacillus subtilis strain WS1A was isolated from a marine sponge from the Saint Martin’s Island area of the Bay of Bengal, Bangladesh. WS1A was cultured on Zobell agar plates (1, 2). It can grow in both marine water and freshwater media. It demonstrated *in vitro* antimicrobial activity against Aeromonas veronii, prevented motile *Aeromonas* septicemia in an Indian major carp species (Labeo rohita) (3, 4), and is considered a promising probiotic candidate. Prior permission was obtained from the Institute of Biotechnology and Genetic Engineering ethical review committee for the animal experiments (approval number IBGE-ERC-005).

WS1A was grown in Zobell broth at 28°C for 24 h, and then the genomic DNA was extracted using the GeneJET genomic DNA purification kit (Thermo Fisher Scientific, USA). The DNA was processed according to the Illumina XT protocol (5). In brief, 1 ng of normalized DNA was fragmented and “tagged” via tagmentation (6). The fragmented DNA was indexed accordingly and subjected to a 600-cycle sequencing protocol using the MiSeq benchtop sequencer (Illumina, Inc.) at 50.0× coverage. Initial identification of bacteria was performed by using the Bacterial Analysis Pipeline v.1.0.4 (7). Removal of sequence adaptors and quality filtering were performed by using Trimmomatic v0.38 and PRINSEQ v0.20.3, respectively (8, 9). The *de novo* assembly and quality evaluation of the assembled draft genome were completed using SPAdes v3.9.0 (10) and QUAST v5.0.2 (11), respectively. Annotation was carried out using the NCBI Prokaryotic Genome Annotation Pipeline (PGAP) (12). Gene prediction was carried out using Prodigal v1.20 and the predicted proteins were searched for similarity against the UniProt protein database using BLASTp v2.10.0 (13), following pathway identification by the KEGG Automatic Annotation Server (KAAS) v2.1. The genome was screened to determine putative antibiotic resistance genes (ResFinder v4.0) (14), plasmids (PlasmidFinder v2.0) (15), virulence factors (VirulenceFinder v2.0) (16), and pathogenicity toward the human host (PathogenFinder v1.1) (17).

The annotated chromosome length, GC content, and *N*_50_ value of the assembled genome were 4,148,460 bp (151 contigs), 43.6%, and 199,148 bp, respectively. The largest and smallest contigs were 433,806 bp and 257 bp, respectively. The open reading frames of the genome were predicted and annotated using the Rapid Annotations using Subsystems Technology (RAST) server (classic RAST FIGfams v70) (18), which showed 333 subsystems and 96 RNA genes.

Genome analyses identified several orthologs of intrinsic genes of a potential probiotic bacterium, such as those encoding proteins involved in the biosynthesis of riboflavin, vitamin B_6_, and amino acids (*ilvD*) and in carbon utilization (*pta*). The genome also codes for antimicrobial peptides such as bacillaene, subtilin, bacillibactin, surfactin, fengycin, bacilysin, and subtilosin A (antiSMASH v5.1.2) (19). No genes coding for putative virulence factors, no plasmids, and no antibiotic resistance genes were identified in the genome using VirulenceFinder v2.0 (16), PlasmidFinder v2.0 (15), and ResFinder v4.0 (14), respectively, with default parameters. The genome sequence information will help to exploit the probiotic potential of this strain.

### Data availability.

The whole-genome shotgun project for B. subtilis strain WS1A has been deposited at GenBank under the assembly accession number JABFHE000000000.1. Raw reads and raw sequencing data are available under the accession number SRR11868367, BioProject accession number PRJNA630208, and BioSample accession number SAMN14828537.
